# Indirect Reciprocity and the Evolution of Prejudicial Groups

**DOI:** 10.1038/s41598-018-31363-z

**Published:** 2018-09-05

**Authors:** Roger M. Whitaker, Gualtiero B. Colombo, David G. Rand

**Affiliations:** 1 Cardiff University, School of Computer Science and Informatics, 5 The Parade, Roath, Cardiff, CF24 3AA UK; 2 Crime and Security Research Institute, Friary House, Greyfriars Rd, Cardiff University, Cardiff, CF10 3AE UK; 3 MIT Sloan School of Management, 30 Memorial Dr, Cambridge, MA 02142 USA; 4 MIT Department of Brain and Cognitive Sciences, 43 Vassar St, Cambridge, MA 02139 USA

## Abstract

Prejudicial attitudes are widely seen between human groups, with significant consequences. Actions taken in light of prejudice result in discrimination, and can contribute to societal division and hostile behaviours. We define a new class of group, the prejudicial group, with membership based on a common prejudicial attitude towards the out-group. It is assumed that prejudice acts as a phenotypic tag, enabling groups to form and identify themselves on this basis. Using computational simulation, we study the evolution of prejudicial groups, where members interact through indirect reciprocity. We observe how cooperation and prejudice coevolve, with cooperation being directed in-group. We also consider the co-evolution of these variables when out-group interaction and global learning are immutable, emulating the possible pluralism of a society. Diversity through three factors is found to be influential, namely out-group interaction, out-group learning and number of sub-populations. Additionally populations with greater in-group interaction promote both cooperation and prejudice, while global rather than local learning promotes cooperation and reduces prejudice. The results also demonstrate that prejudice is not dependent on sophisticated human cognition and is easily manifested in simple agents with limited intelligence, having potential implications for future autonomous systems and human-machine interaction.

## Introduction

Prejudice is a human attitude involving generally negative and unsubstantiated prejudgement of others. When acted upon, this results in wide-ranging behaviours such as sexism, ageism and discrimination against sexual preference^1–3^ through to ethnic, racial, nationalistic and religious extremism^4,5^, with bias and intergroup conflict characterised as a “problem of the century”^6^. Most recently, prejudice has been highlighted in connection to global political events: for example anti-immigration prejudice was a strong correlate of support for Brexit^7^.

The human disposition to categorize others through their group identity creates an opportunity for discrimination^8–10^. As a consequence of in-group formation^11^, which occurs through cultural or biological identification with others, or as a consequence of identity-less strangers mutually cooperating^12^, bias can take hold in two ways. Through *in-group favoritism*^13–16^, people prefer to help fellow group members, while *out-group prejudice*^6,17,18^ represents hostility to those beyond the in-group. These phenomena are easily triggered in human subjects under a wide range of transient experimental conditions^8,13,16,19^. This has contributed to a misperception that positive discrimination to the in-group and negative discrimination to the out-group are inevitable^20^.

Confusion arises between out-group prejudice and in-group favoritism because both concepts potentially reinforce the in-group, but as a consequence of different psychological mechanisms. While in-group favoritism is based on mutual attraction, out-group prejudice discounts the out-group by negatively accentuating differences. In-group favoritism does not depend on negatively biased attitudes, where as prejudice does. From a psychological perspective, this renders in-group favoritism insufficient to model prejudice. Consequently, understanding the separate roles of in-group and out-group discrimination is socially important^6^. In-group favoritism has received significant attention, but the evolution of out-group prejudice has received a more limited explicit focus.

Evolutionary game theory provides a powerful framework to examine the dynamics that can promote discriminatory behaviour^14,15,21–25^. In particular, tag based models^15,26–34^ have shown that spontaneous cooperation can emerge from an agent’s donation being related to whether the recipient’s ‘tag’ is sufficiently similar to their own. Tags are arbitrary symbols upon which discrimination can be made, which must propagate with a behavioral strategy for cooperation to emerge. Groups of individuals can be defined through common tags where the model allows (e.g.^15^). This has established insights into in-group favoritism, particularly that the ability to discriminate between the in-group and out-group can actually promote cooperation, helping to explain why a predisposition toward in-group favoritism have evolved and can be easily triggered^15^.

Beyond tags, alternative models for studying the evolution of in-group favoritism are limited. Fu *et al*.^14^ provide an alternative generalised approach based on evolutionary set theory^11^ that permits out-group as well as in-group interactions. Tag based models generally prohibit this, other than in^33,34^ where although explicit groups are not defined, the model allows individual probabilities of cooperation with dis-similar others to evolve. Fu *et al*.^14^ allow agents to move between sets, with successful sets attracting members and successful strategies gaining imitators. Agents can also differentiate between in-group and out-group strategies and conditions are determined under which preferential in-group cooperation is favoured by selection.

A further relevant consideration is so-called *parochial altruism*^35–39^, where out-group discrimination has mainly been examined under coevolution with in-group favoritism. Both these costly discriminatory behaviours have been proposed as necessary for success in warfare^35^, possibly promoting their coevolution^36^. Parochial altruism is also observed as deeply embedded in human group behaviour^37,39^, although further clarification is needed on the analysis of the selective mechanisms at work in current models^38^.

While prejudice is common, its manifestation is fluid, indicating that culture and cultural evolution^40^ must play an important role in the evolution of bias, through socially transmitted beliefs that help to create and sustain groups. In previous related models, we note that discrimination is considered independently from a group’s identity. Typically, groups of individuals are modelled as a consequence of a common arbitrary tag, and evolution acts upon the agent’s discriminatory strategy in association with that tag. However humans have the capacity to directly identify with a discriminatory attitude as a phenotypic tag in its own right. As such, a discriminatory attitude towards the out-group can provide a common defining feature for a group. In other words, prejudicial (or non-prejudicial) views can act to bind a group and define its boundary. We note that prejudicial feelings towards other groups have been predicted as a consequence of the perceived threat that they pose^41^. Also, at the extremes of group identity, common out-group prejudicial attitudes, from within a larger population, are a particular feature of homophilic attraction and group identity (e.g.^42,43^).

Accordingly, in this paper we introduce and study the evolution of a new abstract class of group, the *prejudicial group*, defined by the common prejudicial disposition of its members towards the out-group. We assume that a population of agents is composed of sub-populations, each denoted *SP*_*t*_, where the agents in *SP*_*t*_ have the common immutable trait *t*. A *prejudicial group* ${G}_{t,\alpha }$ within a sub-population *SP*_*t*_ represents the maximal subset of agents with a common prejudicial attitude (*α*) to the out-group. We use *i* to index the particular parameter values held by an agent *i,* with *t*_i_ indicating *i*’s trait and *α*_i_ indicating *i*’s prejudice level. Therefore *t*_i_ = *t* and *α*_i_ = *α* if and only if agent *i* is a member of *G*_t,α_. An out-group member is any agent not carrying both the prejudicial attitude value *α* and trait *t*. This arrangement gives a simple representation of features such as nationalism, or political, ideological, religious or extremist convictions within a sub-population. People who are less favourable to one out-group tend to be less favourable to other out-groups^44^, and therefore we do not distinguish between them: an agent’s prejudice level *α*_*i*_ is equally applied to all out-groups. The groups ${G}_{t,\alpha }$ partition the sub-population *SP*_*t*_, so that every agent belongs to precisely one group. For experimental purposes we assume *α* ∈ {0, 0.25, 0.5, 0.75, 1}.

Our aim is to observe how *α*_*i*_ evolves with cooperation, and to further understand the conditions that promote or impede *α*_*i*_. We seek to achieve this in a context aligned with observed human behaviour. Across all species, only humans fully engage with indirect reciprocity^23,45^ making it an appropriate cooperative scenario to consider^46^. Indirect reciprocity is commonly examined using the donation game, a special case of the mutual aid game^47^, where agents choose whether or not to donate at cost *c* to a recipient who gains benefit *b* > *c* > 0, without the guarantee of future reciprocation.

Strategies for indirect reciprocity are generally driven by reputation^46,48^, which acts as a currency to judge third party agents who may never be encountered again. While a range of evolutionary approaches to sustaining indirect reciprocity are known^22,46,49,50^, the social comparison of reputation between a donor and the recipient has recently been developed^25^, where the heuristic of donating to those with similar or higher reputation evolves to sustain cooperation. This is of high relevance to prejudice because social comparison is a widespread human disposition^51–55^ that plays a fundamental role in categorization^8–10^ and subsequent stereotyping^18^. Therefore we extend this model^25^ to incorporate prejudicial attitudes against out-group agents, allowing agents to discount the reputation of out-group members by a factor of *α*_*i*_ when considering whether or not to donate.

The model we develop involves 100 agents, which are randomly selected to play the donation game 5000 times, and which constitutes one generation, before evolution of the agents’ strategy. Reputation is central to the model, and *assessment rules* are applied to update a donating agent’s reputation in light of their donation behavior immediately after each donation game. Assessment rules represent social norms, which humans are well-disposed to internalising and perpetuating^56–58^. These enable the judgement of reward and penalty, which are a basis for modelling morality^59^. Because prejudicial groups are defined by the common out-group prejudicial disposition of their members, it is appropriate to model an *in-group reputation* for each agent *i*, denoted ${r}_{i}^{G}$, as well as a *universal reputation* for agent *i*, denoted ${r}_{i}^{U}$, which is the hypothetical reputation in the absence of any prejudice or groups. This approach applies social norms both locally and globally.

Wide-ranging assessment rules have been previously studied^22,60–64^, however standing^62^, with its origins in the work of Sugden^60^, has emerged as one of the dominant approaches because it permits “legitimate shirking”. Here, an agent’s reputation is not reduced when there is a justified basis for defection (e.g., the potential recipient is a defector). We apply a generalised form of standing for both in-group and universal reputations, where a reputation is permitted to range between −5 and +5 in unit steps, as employed in^49^. This choice is based on the analysis conducted in^25^, where the moral conventions of judging, image scoring and standing were compared, allowing evolution to act upon all possible social comparison action rules. These results indicated that either standing or judging are preferential rules, and this comes from their ability to ensure those agents who are limited in their cooperation are not rewarded.

Both the in-group and universal reputations are conceptually simple but require a number of criteria to update them in light of the donation or defection behaviour by an agent *i*. Both types of reputation follow the principles of standing. Specifically, ${r}_{i}^{U}$ and ${r}_{i}^{G}$ are incremented when the donor *i* cooperates. For the universal reputation, if agent *i* defects on agent *j*, whose reputation is of relatively low standing (i.e., ${r}_{j}^{U}$ is lesser than ${r}_{i}^{U}$), then this is deemed legitimate and *i* suffers no penalty to its reputation (i.e., ${r}_{i}^{U}$ remains unchanged). However if *i* defects on agent *j* and this isn’t deemed legitimate (i.e., ${r}_{j}^{U}$ is the same or greater than ${r}_{i}^{U}$) then *i*’s universal reputation is decremented.

Concerning in-group reputation, when *i* and *j* belong to the same group ${G}_{{t}_{i},{\alpha }_{i}}$, the updating of ${r}_{i}^{G}$ is analogous to updating the universal reputation, but through comparing ${r}_{i}^{G}$ with ${r}_{j}^{G}$. However, when *j* is out-group, prejudice comes into play and *i*’s in-group reputation is compared with *j*’s universal reputation as discounted by *i*’s prejudice level. If *i* defects and *j*’s discounted reputation ${r}_{j}^{U}\cdot \mathrm{(1}-{\alpha }_{i})$ is less than ${r}_{i}^{G}$ then this is deemed legitimate by *i*’s in-group and *i*’s in-group reputation remains unchanged, otherwise ${r}_{i}^{G}$ is decremented. Note that in-group reputation may deviate from universal reputation as a consequence of prejudice.

The donation behavior of each agent *i* (i.e., the action rule) is governed by a *social comparison heuristic*, denoted *H*_*i*_ =  (*s*_*i*_, *u*_*i*_, *d*_*i*_, *α*_*i*_, *P*_*i*_, *S*_*i*_). Upon being selected to play, an agent *i* randomly determines its potential recipient *j*, using the probability *S*_*i*_ to determine whether *j* is selected from in-group (with probability 1 − *S*_*i*_ that *i* is selected from an out-group). Variables *s*_*i*_, *u*_*i*_, *d*_*i*_ and *α*_*i*_ allow a donor agent *i* to compare its reputation against that of the potential recipient *j*, and to make the donation decision.

An agent *i* plays a donation game by comparing its reputation against that of *j*, and three outcomes are possible. Assuming *i* and *j* are in the same group, these are similarity $({r}_{j}^{G}={r}_{i}^{G})$, upward self-comparison $({r}_{j}^{G} > {r}_{i}^{G})$, or downward self-comparison $({r}_{j}^{G} < {r}_{i}^{G})$. The reputation ${r}_{j}^{G}$ is replaced with ${r}_{j}^{U}\cdot \mathrm{(1}-{\alpha }_{i})$ in these comparisons when *j* is out-group to *i*. The binary variables from *i*’s social comparison heuristic govern whether or not *i* donates when similarity (*s*_*i*_), upward comparison (*u*_*i*_) or downward comparison (*d*_*i*_) is observed by *i* in respect of *j*. On closure of a generation, a reproductive step conducts natural selection on the social comparison heuristics. Similar to approaches used in a spatial context (e.g.^15,65^), we limit the opportunity for each agent’s reproduction at an evolutionary step to be 10%. This controls potential genetic drift due to selection from within small sub-populations, and the reproductive step is repeated over 50,000 generations, unless otherwise stated. At each reproductive step, if selected to reproduce, an agent *i* chooses another agent’s social comparison heuristic to copy. Based on the Island model^49,66^, copying may be local (i.e., from within the in-group) with probability *P*_*i*_ or from the whole population (with probability 1 − *P*_*i*_). Agent *i* then selects a new social comparison heuristic with chance proportional to the relative fitness of the in-group members or the whole population, while further applying a random mutation to each element of the agent’s new social comparison heuristic, at the rate of 1%^25^. The fitness of an agent is taken as the cumulative difference between the benefits received and costs paid since the previous reproductive step. This genetic reproduction extends that applied in previous work^25^, and follows the general approach of asexual reproduction^49^.

**Figure 1:**
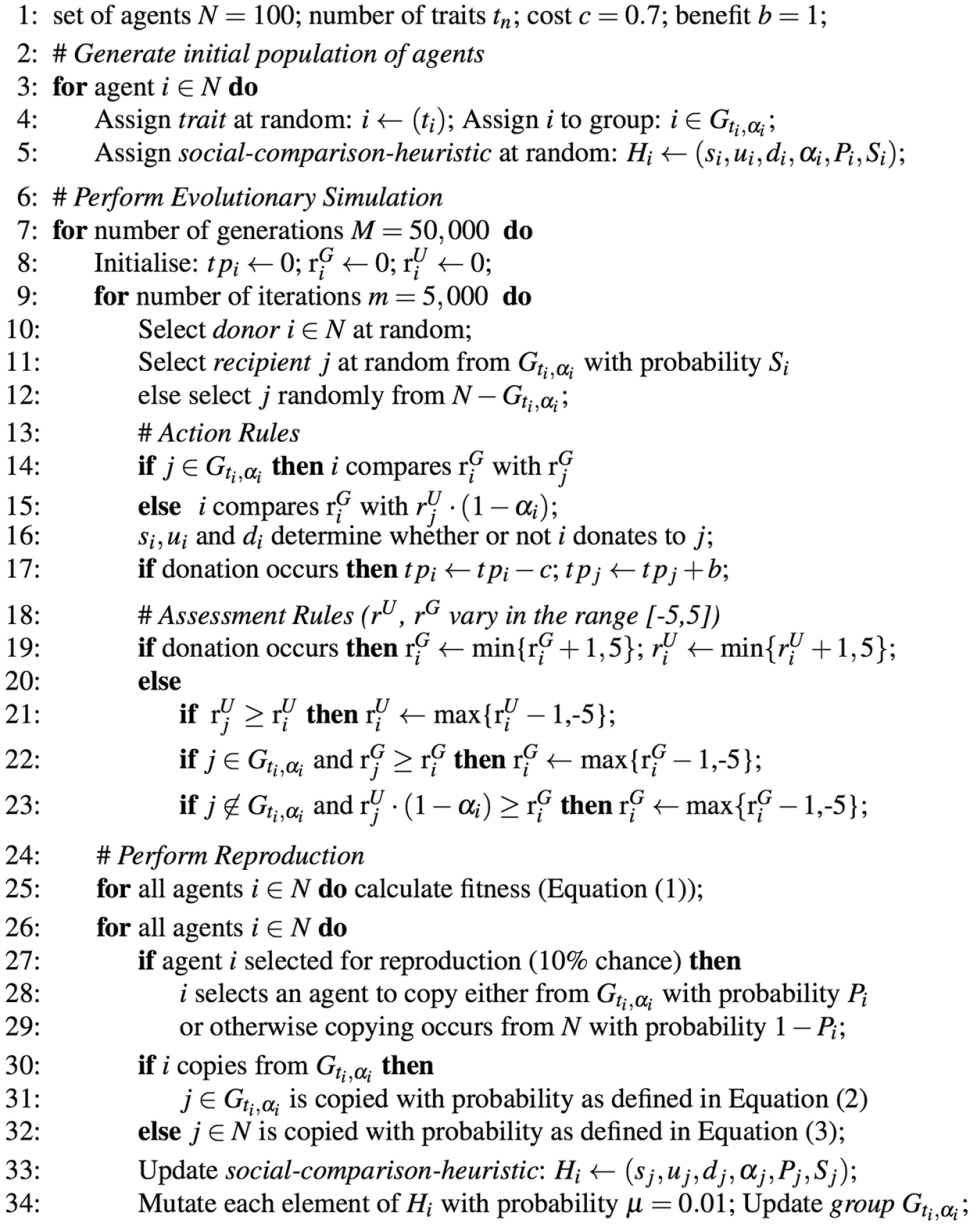
Representation of the pseudocode for simulations, using parameters from Table 1, and supported by further description in the Methods Section.

Note that the reproductive process represents a way in which an agent *i* effectively learns from others, by probabilistic copying, based on the proportional fitness. *P*_*i*_ controls the extend to which this learning is in-group, where only the strategies (i.e., social comparison heuristics) of agents in the same group ${G}_{{t}_{i},{\alpha }_{i}}$ are considered. When *P*_*i*_ is low, agent *i* has a greater chance of learning from beyond its own group, across the wider population. For various experiments *P*_*i*_ and *S*_*i*_ may be exogenously fixed, enabling the influence of these variables to be assessed. A summary of the key parameters is presented in Table 1. The subtle dynamics underlying donation and reputation systems impede formal analysis (such as evolutionary stable strategies), but as in wide ranging studies where this is also the case^15,25,26,65,67^, we employ agent-based simulation. A summary of the pseudocode is also presented in Fig. 1.

**Table 1:** Key parameters of the model for an agent *i*.

Parameter(s)	Description	Role in model	Subject to Natural Selection
*u*_*i*_, *d*_*i*_, *s*_*i*_	Rules for *i*’s donation based on self comparison of the potential recipient *j*’s reputation, while applying prejudice if *j* is out-group	Governs *i*’s donation behaviour	Yes
*α*_*i*_	Prejudice level of *i*	Defines the level by which *i* reduces the universal reputation of out-group agents. Used to define commonality with others to create an in-group ${G}_{{t}_{i},{\alpha }_{i}}$	Yes
*t*_*i*_	Fixed trait held by *i*	Controls the sub-population to which *i* belongs	No
${G}_{{t}_{i},{\alpha }_{i}}$	The in-group to which *i* belongs, defined by agents with both the same fixed trait and prejudice level as *i*	Defines the in-group boundary for *i*, determining whether or not *i* applies a prejudicial view of other agents	Yes, as a consequence of *α*_*i*_ evolving
*S*_*i*_	The probability that *i* plays the donation game with a randomly chosen member of the in-group, rather than from an out-group	Governs how well-mixed the interactions are between agents in different groups	Optional – can evolve (Fig. 2) or exogenously fixed (all other Figs.)
*P*_*i*_	The probability that *i* reproduces by selecting a social-comparison heuristic from in-group, rather than from the whole population	Governs how the in-group focused agents are, from learning by copying the social comparison heuristics of others	Optional – can evolve (Fig. 2) or exogenously fixed (all other Figs.)
${r}_{i}^{U}$	Universal reputation for *i*	Status of the donation behaviour of *i*, based on generalised standing, assuming prejudice is not legitimate when considering the recipient’s reputation	No
${r}_{i}^{G}$	In-group reputation for *i*	Status of the donation behaviour of *i*, from the in-group perspective, which assumes that prejudice is applicable to out-group reputation	No
*tp*_*i*_	total payoff for *i*	Accumulates costs and benefits from making and receiving donations	No

**Figure 2:**
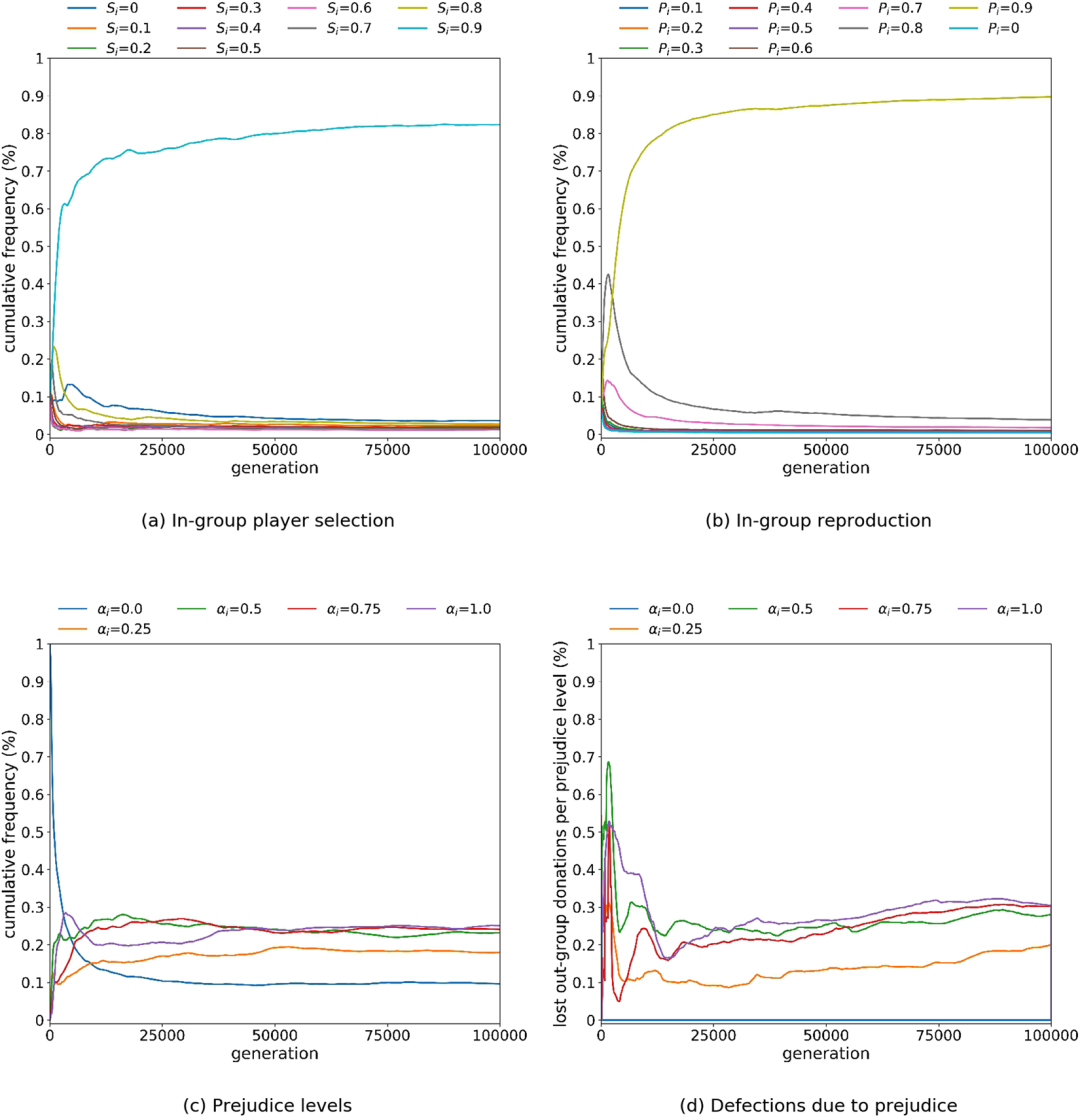
Evolution of 100 agents with 5 equal-size sub-populations over 100,000 generations. Initial conditions: *d*_*i*_, *s*_*i*_, *u*_*i*_, *S*_*i*_ and *P*_*i*_ are randomly selected and *α*_*i*_ = 0 ∀*i*. *S*_*i*_ and *P*_*i*_ are permitted to evolve in the range {0, 0.1, …, 0.9}. The cumulative proportion of agents by values of *S*_*i*_, *P*_*i*_ and *α*_*i*_ is shown in (**a**), (**b**) and (**c**) respectively. (**d**) Shows the cumulative proportion of out-group defections that occur as a consequence of prejudice. In the absence of prejudice, this additional proportion of out-group donations would have occurred.

Note that these parameters populate an agent’s social comparison heuristic, denoted *H*_*i*_ = (*s*_*i*_, *u*_*i*_, *d*_*i*_, *α*_*i*_, *P*_*i*_, *S*_*i*_). Natural selection occurs with respect to *P*_*i*_ and *S*_*i*_ in Fig. 2, but *P*_*i*_ and *S*_*i*_ are exogenously fixed in all other Figures, as a means to consider scenarios where these variables are set by the external context.

## Results

Firstly we investigate the emergence of prejudicial groups when all variables in an agent’s social comparison heuristic are permitted to evolve. We partition the population into 5 equal sub-populations and consider evolution over 100,000 generations. Figure 2 shows how agents become in-group focussed in three dimensions. Natural selection of players focuses interactions on the in-group (high *S*_*i*_), accompanied with the emergence of prejudice (presence of high *α*_*i*_), which occurs within 10,000 generations. At the same time, in-group learning rapidly emerges (high *P*_*i*_) - aligning a choice of strategy with in-group selection.

Prejudice enters the population through mutation. Agents with strategies that discount the out-group reduce their risk of making donations that may not be reciprocated. As the presence of prejudicial groups increases, agents with a non-prejudicial disposition or an out-group focus for interaction become more exposed to free-riding recipients, because reciprocation has a limited chance of occurring from prejudicial out-groups. This further promotes prejudice, with in-group learning (*P*_*i*_) ensuring that agents avoid risking a strategy that has been successful in the context of a different group’s behaviour. Interestingly, although considerable prejudice is evident, cooperation rapidly evolves, and the social comparison heuristic of *d*_*i*_ = 0, *s*_*i*_ = 1 and *u*_*i*_ = 1 dominates, consistent with previous research^25^.

Prejudice and cooperation are often perceived as antagonistic forces. However the results show how cooperation coevolves with prejudice, specifically that prejudice discounts cooperation from the out-group and restricts it to the in-group. Figure 2 also shows that this doesn’t come without a cost. Although *S*_*i*_ evolves to preference in-group selection of players for the donation game, opportunities to receive donations from an out-group are lost (Fig. 2(d)), with higher prejudicial groups proportionally contributing the most to this loss.

The experiments reported in Fig. 2 allow natural selection to act on all variables in an agent’s social comparison heuristic. However, two variables in particular are open to exogenous influence: the tendency for in-group interaction (*S*_*i*_) and the extent of in-group learning (*P*_*i*_) verses global learning. These factors abstractly reflect the pluralism of a society, being influenced by issues beyond the individual, such as social policy, government, historical conflict, culture, religion and the media. To further investigate the nature of prejudicial groups, we proceed by considering *S*_*i*_ and *P*_*i*_ as external variables that are set by context (i.e., specified and fixed by the experiment) rather than free to be chosen by natural selection. This allows observation of prejudicial groups assuming different pluralistic scenarios. In particular, it allows us to understand the role of interactions and social learning as variables that contribute to the emergence and mitigation of prejudice.

### Cooperation, prejudice and pluralism

**Figure 3:**
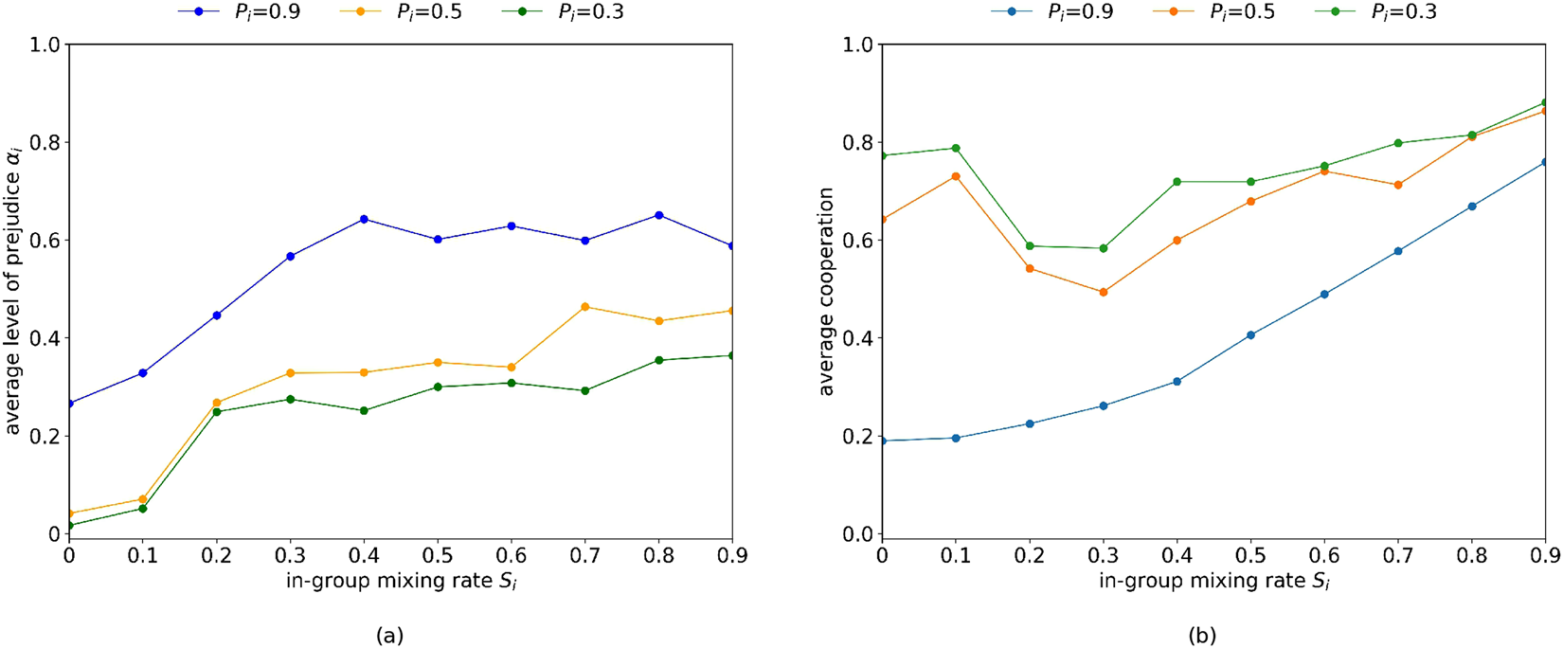
(**a**) The average level of prejudice vs. in-group mixing for different in-group learning rates and (**b**) the average cooperation vs. in-group mixing for different in-group learning rates. *S*_*i*_ and *P*_*i*_ are a-priori fixed for all agents, and results represent the average of 10 randomly seeded runs, each conducted for 50,000 generations. Initial configuration involves *α*_*i*_ = 0, ∀*i*. Mutation applied at the rate *μ* = 1/100 to each variable *d*_*i*_, *s*_*i*_, *u*_*i*_ and *α*_*i*_. Five equally sized sub-populations are assumed.

Figure 3 shows how prejudice and cooperation is sustained in terms of *P*_*i*_ and *S*_*i*_ when they remain fixed during the simulation, for all agents. This assumes that the extent of pluralism, in terms of interaction and learning, is set by the external context. Lower levels of in-group mixing are necessary to attain lower levels of average prejudice (Fig. 3a), and this is most effective when the chance of global learning is high (low *P*_*i*_). Cooperation generally benefits from higher in-group mixing (Fig. 3b), which is compounded by increased global learning (low *P*_*i*_). High in-group mixing reduces the opportunity for cooperation to be diminished by prejudice. The exception here are extreme circumstances (e.g., *S*_*i*_ < 0.2 and *P*_*i*_ ≤ 0.5) where there is limited opportunity for a prejudicial group to gain utility from in-group interactions. The relationship between cooperation and prejudice is further mapped throughout the results.

### Small mutations are sufficient to cause prejudicial groups

Susceptibility to prejudice can be considered by the extent to which a small chance of mutation in an agent’s *α*_*i*_ value, given an initially non-prejudicial population (∀*i*, *α*_*i*_ = 0), is sufficient to trigger more widespread prejudicial attitudes in conditions where prejudice can emerge (Fig. 3a, *S*_*i*_ ≥ 0.2). This can be examined by considering a population with low pluralism, such as one with little out-group interaction (e.g., *S*_*i*_ = 0.8) and high in-group learning (e.g., *P*_*i*_ = 0.9), consistent with Leimar *et al*^49^.

**Figure 4:**
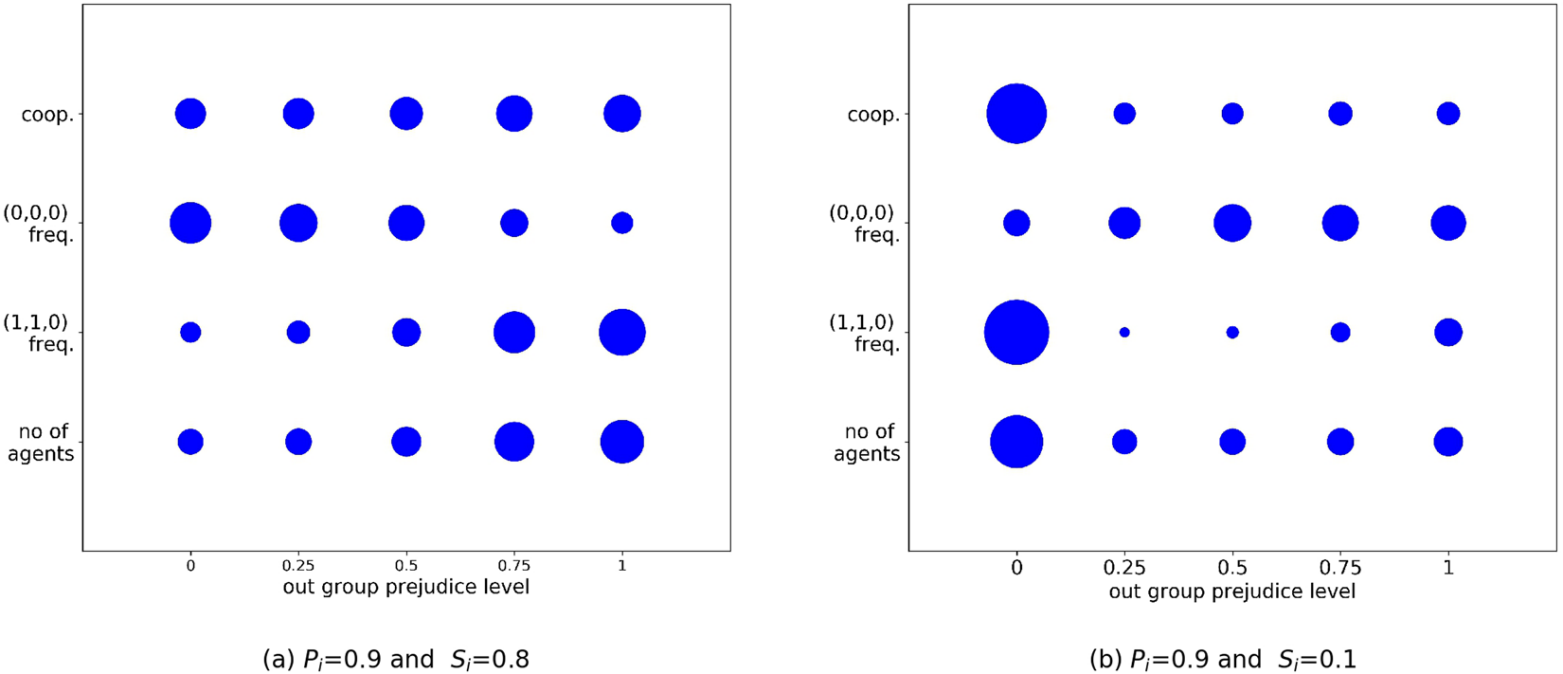
The relative cumulative frequency of agents’ characteristics by prejudice level. Characteristics concern number of agents (no of agents); instances of the dominant cooperative social comparison heuristic *s*_*i*_ = *u*_*i*_ = 1 and *d*_*i*_ = 0 ((1,1,0) freq.); instances of the defection social comparison heuristic *s*_*i*_ = *u*_*i*_ = *d*_*i*_ = 0 ((0,0,0) freq.); instances of cooperation (coop). All simulations assume five sub-populations (i.e., 5 traits). (**a**) *P*_*i*_ = 0.9 and *S*_*i*_ = 0.8; (**b**) *P*_*i*_ = 0.9 and *S*_*i*_ = 0.1, fixed for all agents over 50,000 generations. The circles represent the relative proportions of agents with those characteristics across ten randomly seeded runs. Starting configurations involve all agents having zero prejudice (∀*i*, *α*_*i*_ = 0).

We consider these fixed values for *S*_*i*_ and *P*_*i*_. All agents are initially prejudice free (*α*_*i*_ = 0,∀*i*) and Fig. 4a shows the resultant prejudicial characteristics. Mutation at a rate of *μ* = 1/100 creates prejudicial individuals relatively infrequently, but when a small number of such individuals occur with the same prejudice level, a non-trivial group can establish itself. Prejudicial individuals prosper by building a strong in-group reputation, while retaining resources rather than donating to the out-group. This limits their universal reputation and limits the extent of donations received from the out-group. When there is dominance of in-group interactions (e.g., *S*_*i*_ = 0.8), this is not an impediment to their payoff and it also reduces their costs.

Even when in-group mixing is lower (e.g., *S*_*i*_ = 0.4) it is still sufficient for prejudicial groups to establish themselves. Cooperation through in-group interaction involves social comparison heuristics with *s*_*i*_ = *u*_*i*_ = 1 and *d*_*i*_ = 0 emerging as prevalent, which limit an agent’s exposure to those of lower standing within the group, offering relative protection against shirkers, consistent with previous findings^25^. Non-prejudicial groups become relatively disadvantaged when prejudice is present, as they are susceptible to making out-group donations without reciprocation. This leads to the emergence of defection (i.e., *s*_*i*_ = *u*_*i*_ = *d*_*i*_ = 0) as the preferential strategy for these groups.

### High levels of out-group interaction suppress prejudice irrespective of global learning

When out-group interactions are high (i.e., *S*_*i*_ is fixed as low), agents become increasingly dependent on the out-group for donation of resources, and universal reputation becomes important. Agents discriminating through prejudice are less likely to make out-group donations, restricting their opportunity to build a high universal reputation that is attractive to out-group donors, impeding the payoff for prejudicial group members. This gives advantage to lower prejudice agents.

Figure 4b shows an example of the evolution of groups under high rates of out-group interaction (*S*_*i*_ = 0.1). In contrast to Figure 4a, which is exactly equivalent other than for the setting of *S*_*i*_ (*S*_*i*_ = 0.9), Figure 4b shows that extensive out-group interactions are sufficient to promote non-prejudicial groups that are more cooperative.

### Even when opportunities for mixing are limited, prejudice can be mitigated through learning

Societies which are in-group focused with their interactions can still mitigate prejudice through learning from the wider population. Figure 3a shows that for all *S*_*i*_, when out-group learning increases (*P*_*i*_ is reduced), the average frequency of prejudice is diminished. This exposes agents to a wider range of strategies for selection. However reducing *P*_*i*_ also results in a greater variance in prejudice level frequency (S.D. in the range [0.3, 0.4] when *P*_*i*_ = 0.3, *P*_*i*_ = 0.5 and *S*_*i*_ ≥ 0.2; S.D. in the range [0.003, 0.076] when *S*_*i*_ < 0.2; S.D. in the range [0.071, 0.18] when *P*_*i*_ = 0.9). This is consistent with the population structure having a lesser effect on impeding genetic drift^49^, resulting in sporadic instances of highly prejudicial agents. This occurs alongside strategies in lower prejudice groups that are successful through maintaining a high universal reputation.

### Widespread prejudice is challenging to reverse: trait diversity helps

**Figure 5:**
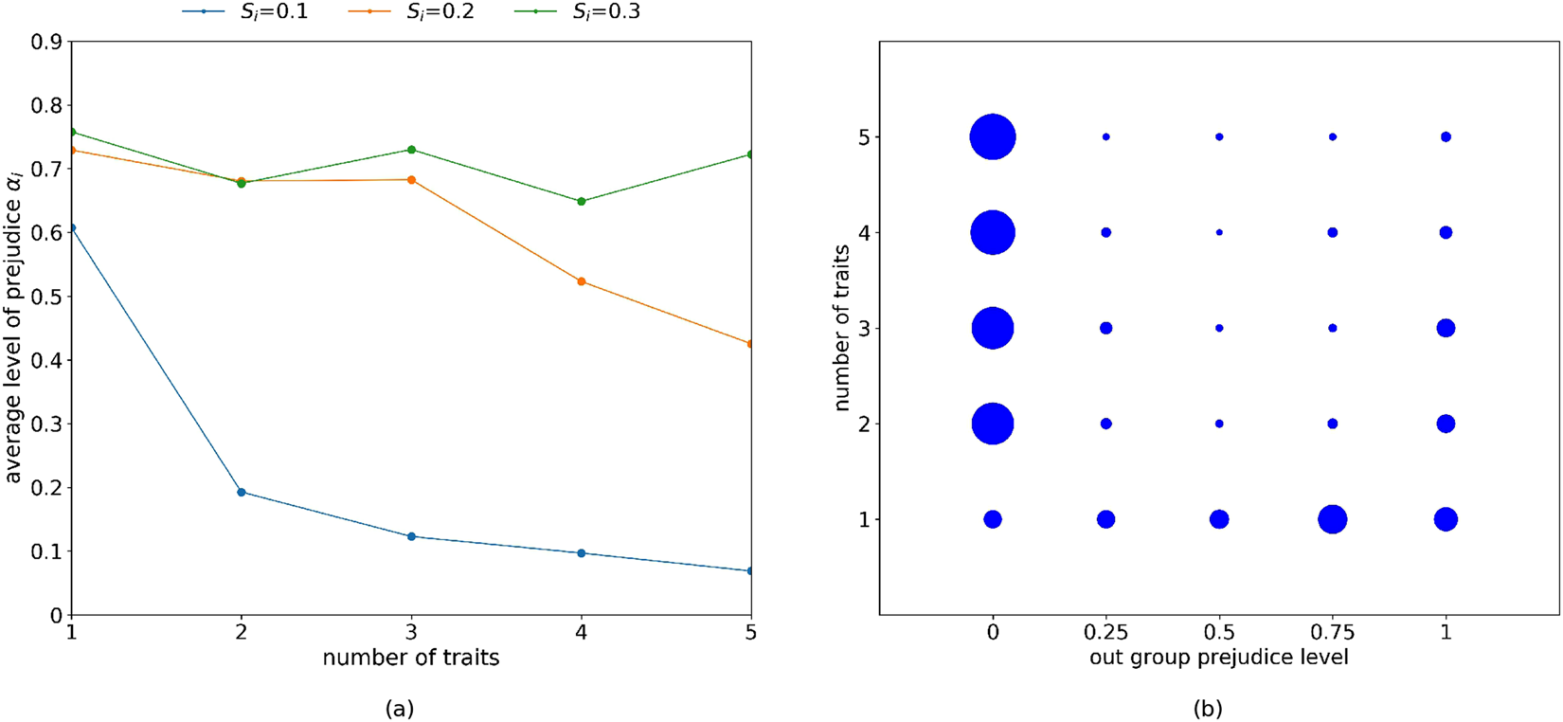
(**a**) Average level of prejudice as a function of the number of traits (sub-populations) with *P*_*i*_ = 0.5 after 50,000 generations. (**b**) The cumulative distribution of instances of agent’s prejudice level by number of fixed traits. The circles represent the relative cumulative frequency over ten randomly seeded runs with *P*_*i*_ = 0.5 and *S*_*i*_ = 0.1, using 50,000 generations per run. The starting configuration involves all agents being fully prejudicial (*α*_*i*_ = 1, ∀*i*), and equally distributed over the number of sub-populations involved.

For a fully prejudicial population, we consider the conditions required to reverse prejudice, focusing on the role of fixed traits as provided by sub-populations. In the initial population we assume that all agents are entirely prejudicial (*α*_*i*_ = 1 ∀*i*) and we explore to what extent prejudice diminishes when the number of fixed traits (i.e., sub-populations) is varied, as shown in Figure 5. We exogenously control for *S*_*i*_ and *P*_*i*_. From previous experimentation (Figure 3) prejudice mitigation is most likely to occur under conditions of high out-group mixing (e.g., *S*_*i*_ < 0.2). Figure 5a shows that as the number of fixed traits increases, the average level of agent prejudice decreases, but this requires very high out-group mixing (*S*_*i*_ = 0.1). Figure 5b shows the cumulative frequency of prejudicial attitudes as the number of fixed traits increase.

Throughout, we evenly split in-group and global learning (*P*_*i*_ = 0.5) for all agents. A mutation rate of *μ* = 1/100 is adopted, being sufficient to trigger the infrequent coexistence of non-prejudicial agents, which form a non-trivial group. When these conditions are present with a single sub-population (i.e., one fixed trait), members of the non-prejudicial group are initially unable to receive donations from any out-group interactions. This constraint is removed when two sub-populations (i.e., two fixed traits) are introduced.

This effect generalises: if we consider a fixed number of total non-prejudicial agents at a particular point in time, as the number of fixed traits (i.e., sub-populations) increase, the non-prejudicial agents can occur with different traits. Therefore the number of non-prejudicial out-group members increase. This means that each non-prejudicial agent has a greater chance of receiving a donation from a non-prejudicial out-group member, increasing the payoff for non-prejudicial agents. As a result, increasing the number of traits promotes lower prejudice.

### Prejudice emerges more easily when group sizes are imbalanced

Previous work concerning the analysis of intolerance under economic stress^68^ identified that it is easier for intolerance to emerge in minorities (i.e., when groups have different sizes). This involved consideration of the leading eight strategies^50^ over two groups. We investigate the extent to which difference in group size has a similar effect in the context of prejudicial behaviours.

**Figure 6:**
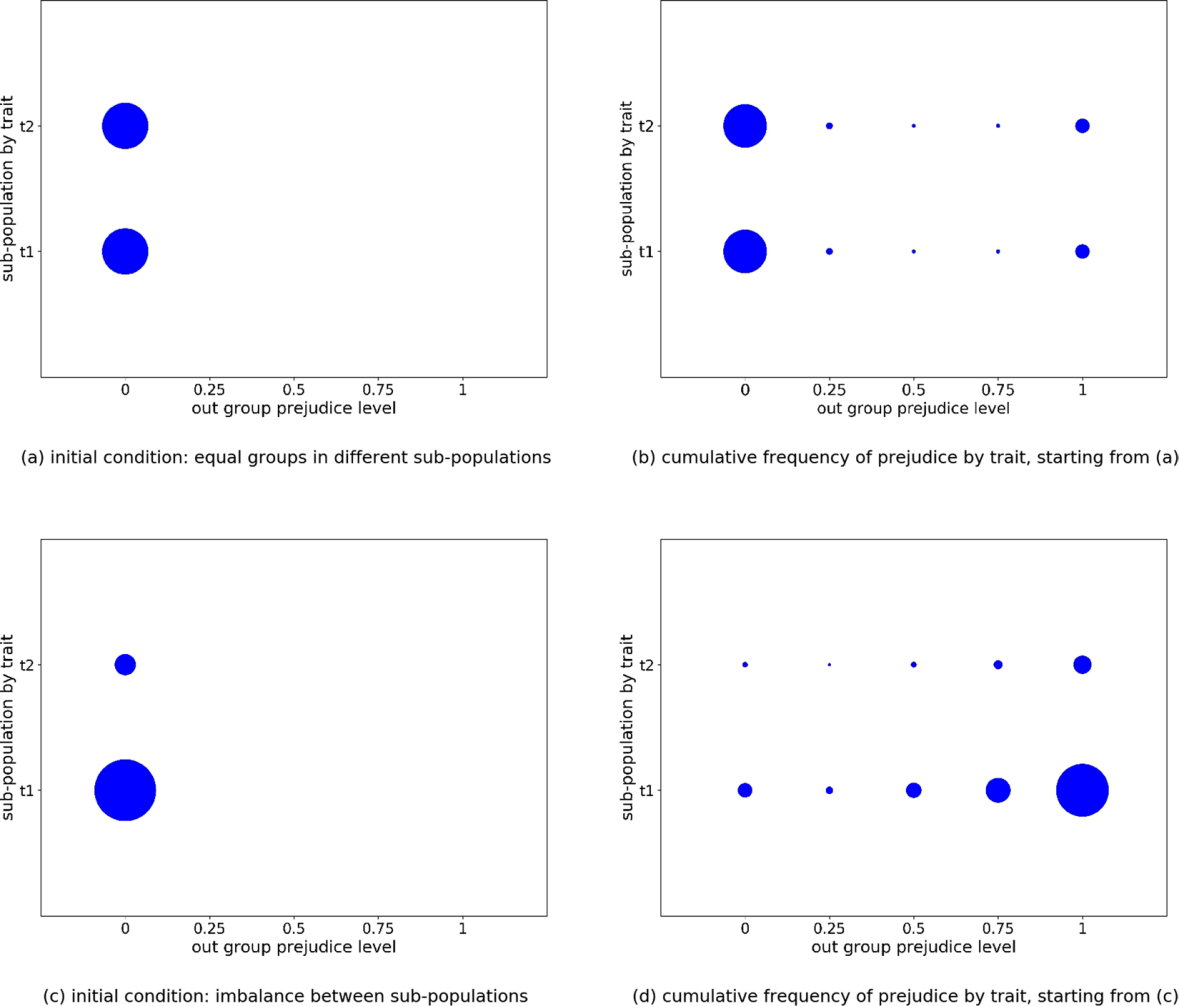
The relative cumulative frequency of agent’s prejudice levels by trait, in a population of 100 agents and two traits. *P*_*i*_ = 0.5 and *S*_*i*_ = 0.5 are fixed for all agents, with simulations carried out using 50,000 generations. Initially all agents *i* have *α*_*i*_ = 0. The circles represent the relative proportions of agents with those particular characteristics, accumulated over ten randomly seeded runs (see (**b**) and (**d**)). Two alternative starting configurations are employed: an equal split between sub-populations (see (**a**)) and a 90/10 imbalance between sub-populations (see (**c**)).

In Figure 6 we examine the evolution of two non-prejudicial groups, each involving a different fixed trait. Using two scenarios, we compare the effect of varying the initial group size, evenly splitting the agents in one scenario and splitting them 90/10 in the other. Group evolution is particularly sensitive to the extent of in-group learning (*P*_*i*_), and a degree of out-group interaction has to be present for effects to be observed. Figure 6 shows that when *P*_*i*_ allows a mix of learning from the in-group and the wider population (e.g., *P*_*i*_ = 0.5), imbalance in group size leads to significantly greater prejudice. This is because any economic advantage from being prejudicial has a greater chance of been copied in-group in a smaller group, purely as a consequence of the smaller group size. Consequently prejudicial views rapidly spread in the smaller sub-population, creating prejudicial groups. The presence of global learning then promotes prejudice in the larger sub-population involving the other trait. This is not seen when the initial sub-groups have the same size.

### In-group mixing and global learning both promote cooperation, but with opposing implications for prejudice

**Figure 7:**
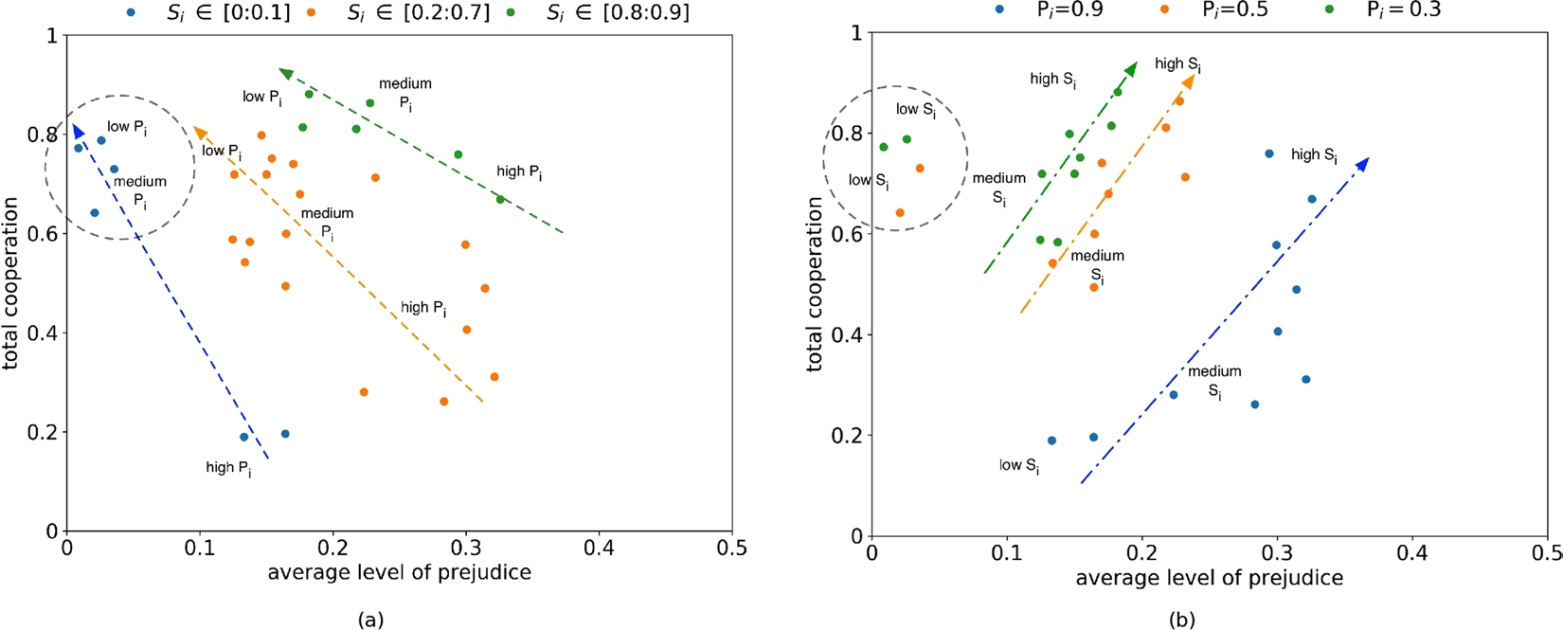
Total cooperation as a function of the average level of prejudice. Calculations are the result of 10 randomly seeded runs. Note that (**a**) and (**b**) show the same data points, but referenced by different parameters. (**a**) For fixed values of *P*_*i*_ (*P*_*i*_ = 0.3, 0.5, 0.9), the effects of varying *S*_*i*_ are indicated. (**b**) For low, medium and high values of *S*_*i*_, the effects of varying *P*_*i*_ are indicated.

Other than for the extreme circumstances (e.g., *S*_*i*_ < 0.2 and *P*_*i*_ ≤ 0.5) where there is limited opportunity for a prejudicial group to gain utility from in-group interactions, attaining cooperation invokes prejudice in some form. We explore this by exogenously controlling for *S*_*i*_ and *P*_*i*_, and examining how prejudice and cooperation co-evolve. Figure 7 shows cooperation as a function of prejudice from two perspectives. Figure 7a shows that increasing in-group interactions promotes cooperation while also increasing prejudice and Figure 7b shows that greater levels of global learning promote cooperation, while also reducing prejudice.

These results implicitly show the roles of *S*_*i*_ and *P*_*i*_: in combination, they provide a trade-off in how cooperation can be achieved and show the role of interaction and learning in sustaining group attitudes. In terms of cooperation, high in-group mixing (high *S*_*i*_) can be compensated for through high levels of out-group learning (high *P*_*i*_), and vice-versa. For example consider a total cooperation level of 0.7 in Figure 7b. This can be achieved with high out-group mixing (e.g., *S*_*i*_∈[0, 0.1]) and medium in-group learning (e.g., *P*_*i*_ = 0.5), which is accompanied by low prejudice levels (*α*_*I*_ ∈ [0, 0.1]). Similar levels of cooperation can be attained with medium in-group mixing (e.g., *S*_*i*_ ∈ [0.2, 0.7]) and low in-group learning (e.g., *P*_*i*_ = 0.3), which is accompanied by medium prejudice levels (*α*_*i*_ ∈ [0.3, 0.4]). Furthermore, we can achieve the same cooperation with high in-group mixing (e.g., *S*_*i*_ ∈ [0.8, 0.9]) and high in-group learning (e.g., *P*_*i*_ = 0.9), which is accompanied by high prejudice levels (*α*_*i*_ ∈ [0.6, 0.7]) which reflect the in-group focus.

Note that all results involve a cost-to-benefit (*c*/*b*) ratio of 0.7. This is chosen based on analysis of the evolution of cooperation and social comparison^25^. This *c*/*b* ratio has been adopted because it is relatively conservative, in the sense that donation is a relatively costly action. Using a lower *c*/*b* ratio means that at the reproduction stage, less impact from defectors is apparent, because lower costs are incurred from donation to them. Therefore prejudicial strategies, that seek to benefit by not donating, provide less of an advantage. This means that alternative lower *c*/*b* ratios result in lower prejudice overall, if applied in this model.

## Discussion

The model we have introduced allows computational exploration of how attitudes towards the out-group, not just behaviours, are sustained and evolve. In particular, the results provide insight into the origins of prejudicial groups as a consequence of natural selection. The model addresses the commonality of prejudicial attitudes as a phenotypic tag that identifies an ideological group. This allows a simple representation through which discriminatory (or non-discriminatory) groups may emerge within a sub-population, enabling modelling of concepts such as nationalism. The model complements previous work^68^ that has analytically studied the emergence of intolerance based on the leading eight donation strategies.

Our general approach involves an agent’s prejudice creating a differential between the reputations of the in-group and those of the out-group. In addition, prejudice provides a basis for an “in-group” to form in the first place, from a common prejudicial disposition. This is a new relationship between out-group discrimination and in-group formation, which is a key aspect of the model, as seen in elements of human behaviour. Note that in-group favoritism could also take hold in prejudicial groups. However, to isolate factors, we have considered the effects of a negative prejudicial bias, but additionally, a positive reputational bias aligned with in-group favoritism could be added. This would further promote the differential between in-group and out-group reputation. Considering the effects of in-group favoritism in prejudicial groups, beyond creating a reputation differential, is possible future work.

The model is based on indirect reciprocity^22,49,62,69^, a form of cooperation that strongly identifies with human behavior as compared to most other species. Indirect reciprocity is generally sustained by agents acting in response to the reputation of a third party^23^. In our model, agents undertake self-comparison of their reputation against that of the potential recipient, whose reputation is discounted by prejudice if they are out-group. Strategies for donation evolve based on self-comparison, alongside the evolution of an agent’s prejudice level, and an agent’s variables concerning the probabilities of in-group interaction and in-group (verses global) learning. At face value, the action rules that we adopt, based on social comparison, differ from the seminal work identifying the “leading eight”^50^. However in previous work we identified their close relationship with our approach^25^. The social norms (assessment rules) we employ are based on a generalisation of standing^60,62^, that are effective in supporting cooperation^25^. However, we note that alternative assessment rules, particularly within the context of extreme prejudicial groups, could be applied within our overall modelling framework.

While in-group favoritism has been studied through explicit models^14^ and as a consequence of the “green beard” effect in tag-based models^15,26–32^, out-group prejudice has received little explicit investigation, with it being easily conflated with in-group favoritism. To the best of our knowledge, only in^33,34^ are tag-based models presented that involve parameters to discount others based on dis-similarity, although prejudicial groups are not defined. Assignment errors often feature in tag-based (and other) models, which generally hinder the evolution of global cooperation levels and cooperation between individuals with different tags. It is possible that prejudice could be expressed through larger assignment errors, but forming groups on that basis would require them to be public. However this is an interesting possible future research direction.

The results in this work allow us to observe how prejudicial groups and cooperation co-evolve. Prejudice and cooperation are often considered to be opposing forces. However we find that they are concurrently sustained, but with significant structural consequences for the population. Prejudice directs cooperation into “islands”^49^ represented by groups holding the same out-group attitude. Thus prejudicial groups promote assortment with potential cooperators^70^, or at least reduce the risk of assortment with those of a perceived threat to defection^41^. While prejudice acts to reduce the risk of donating to groups that may not reciprocate, cooperation evolves in-group. This is combined with agents preferring to play the donation game in-group, and with learning at the evolutionary step predominantly copying in-group strategies, consistent with observations from the origins of the human population (e.g., *P*_*i*_ = 0.9)^49^. Also consistent with previous findings^25^, the dominant heuristic of donating to those with similar or greater reputation levels sustain cooperation emerges in-group. Although prejudice results in lost opportunities to receive out-group donations, this is mitigated through in-group cooperation.

Beyond the economics of payoff, it is important to note that in-group cooperation in lieu of cooperation across the wider population can have significant negative societal implications, occuring as a consequence of the resultant disconnected social network structure. When cooperation is restricted in-group, such groups tend to become increasingly isolated, interacting and learning predominantly from their own members. This disrupts social and cultural connectivity, supporting the “filter bubble” problem where isolated in-group communication affirms and reinforces the group’s own perspective without exposure to wider discourse^71,72^. Structurally, an in-group focus can also diminish the potential benefit from weak ties^73^, which impedes a wide range of societal and economic issues. For example, individuals may become restricted in their opportunity to create or exploit social capital from bridging^74^, limiting social mobility for group members^75^. More generally, the diversity of individuals’ relationships is strongly correlated with the economic development of communities^76^. In the context of organisational innovation, actors who can exploit weak ties have a greater source for ideas^77^, thereby exploiting diversity. We also note that strong external cultural factors can contribute to the tendency for agents to engage in out-group interaction and global learning, which may be slow to change, effectively being fixed over long durations.

Analysis of the leading eight donation strategies under two sub-populations^68^ shows that once intolerance invades one (or both) sub-populations, it becomes difficult to counter. Consistent with this, our results highlight that random instances of prejudice in agents are sufficient for prejudicial groups to become more widespread. After prejudicial groups become manifested in a sub-population, prejudicial attitudes spread as a means to prevent exploitation from the out-group. As a consequence, reversing prejudice is a significant challenge, and multiple factors have influence. Firstly, sub-population diversity increases the opportunity to counter prejudice, enabling out-group cooperation with non-prejudicial groups. Also societies based on high levels of out-group interaction and high levels of global learning provide mitigation. Critically, we also find that in-group mixing and global learning both promote co-operation, but with opposing implications for prejudice. This is the first known characterisation of such a relationship. Notably high levels of cooperation are possible, but with completely different interaction and learning behavior from agents.

An interesting feature of this study concerns the minimal cognitive ability of the agents involved. This decouples prejudice from the psychological and social capabilities that are distinctive in humans^78–80^, and through which prejudice is frequently explained in society. This is significant because research^10,81–85^ on how inter-group contact leads to attitude change is dependent on sophisticated cognition. In particular, the nature of contact through relationships, as perceived through higher order cognition, predicts prejudice in humans^86–88^. However, our model shows that prejudicial groups can easily manifest themselves through natural selection, as applied to agents with primitive cognitive abilities. Furthermore, we note that the pluralistic nature of the environment, in terms of embedded dispositions for out-group mixing, global learning, and the diversity of sub-populations, significantly influence how prejudicial groups evolve.

The findings of our research are also interesting in the context of machines and autonomous systems^89^, in particular for scenarios involving one-shot interactions and learning, where collectively, devices need to sustain cooperation by periodically benefiting from the resources of others, while not necessarily encountering them again. Examples of these scenarios are currently being considered in the context of future communications^90,91^ and may also emerge in future human-machine interaction^92^. Our study highlights the scope for prejudice to emerge in populations of autonomous machines that have simple cognitive abilities, such as being driven by local interactions and the assessment of others. This reaffirms that the distributed collective intelligence of machines is also a social endeavour, and it is potentially susceptible to prejudicial phenomena as seen in the human population.

## Methods

We extend a framework concerning the evolution of indirect reciprocity from the social comparison of reputation^25^. The model involves a population of 100 agents, composed of sub-populations, each denoted *SP*_*t*_, where the agents in *SP*_*t*_ are given a common immutable trait *t*. Each agent belongs to precisely one sub-population (see Agents and Groups Section). We vary the number of sub-populations for different experiments. Agents are randomly selected to play the donation game (see Action Rules Section), and the donor agent’s reputations are updated (see Assessment Rules Section). After 5000 donation games, which constitutes one generation, natural selection is performed (see Selection and Reproduction Section). This cycle is repeated for 50,000 generations unless otherwise stated. All results represent an average of 10 randomly seeded runs. We assume that all agents commence with zero prejudice (*α*_*i*_ = 0), unless otherwise stated. Information on accessing data supporting the results is available^93^. A summary of the related pseudocode is presented in Figure 1.

### Agents and Groups

Each agent *i* is represented by a *social comparison heuristic* denoted (*s*_*i*_, *u*_*i*_, *d*_*i*_, *α*_*i*_, *P*_*i*_, *S*_*i*_). These are acted upon by evolution at the end of each generation, and they govern the donation and reproductive behavior of each individual. Each agent *i* remains in its original sub-population throughout the simulation, but may move between the prejudicial groups within that sub-population, as a consequence of the reproductive step.

Binary variables *s*_*i*_, *u*_*i*_ and *d*_*i*_ concern an agent’s action rules, which are well-understood in isolation^25^. These variables (action rules) are randomly initialised. *α*_*i*_ represents the agent’s out-group prejudice level, where *α*_*i*_ ∈ {0, 0.25, 0.5, 0.75, 1}. The prejudice level for an agent *i* is denoted *α*_i_. We set *α*_*i*_ = 0, for all agents *i,* at the start of each generation, unless otherwise stated. *S*_*i*_ and *P*_*i*_ range in the set {0, 0.1, …, 0.9} unless the experiment fixes *S*_*i*_ and *P*_*i*_. *t*_i_ denotes the particular trait of the sub-population to which *i* belongs.

A *prejudicial group* ${G}_{t,\alpha }$ is the maximal subset of agents with the same trait *t* and prejudice level *α*. Therefore prejudicial groups partition each sub-population. An out-group member to ${G}_{t,\alpha }$ is any agent not carrying both the prejudicial attitude *α* and trait *t*. Agent *i* belongs to group *G*_t,α_ if and only if *t*_i_ = *t* and *α*_i_ = *α*.

The variable *S*_*i*_ controls the selection of *i*’s potential recipient *j* for each donation game, determining the chance that *j* is selected from in-group. *P*_*i*_ determines the chance that *i*’s social comparison heuristic evolves based on the in-group (referred to as in-group learning), as compared to evolutionary influence from the whole population^49^. In numerous experiments we keep *S*_*i*_ and *P*_*i*_ fixed, to understand their effect on the evolution of prejudicial groups.

Each agent maintains two reputations that are used to represent *i*’s reputation from an in-group perspective (${r}_{i}^{G}$) and a universal perspective that assumes no prejudice (${r}_{i}^{U}$). Reputations vary between −5 and +5 in integer steps^25,49^.

### Action Rules

In each generation we perform 5000 random agent selections where each selected agent plays the donation game. Within a particular generation, assume that agent $i\in {G}_{{t}_{i},{\alpha }_{i}}$ is selected to play with *j*. The probability that *j* is selected from the in-group is *S*_*i*_. Otherwise *j* is selected from the out-group. Whether or not *i* donates is governed by *s*_*i*_, *u*_*i*_ and *d*_*i*_, upon *i* comparing its reputation with that of *j*. If *j* is in-group ($j\in {G}_{{t}_{i},{\alpha }_{i}})$ then *i* compares ${r}_{i}^{G}$ with ${r}_{j}^{G}$. Otherwise, when *j* is out-group, *i* compares ${r}_{i}^{G}$ with ${r}_{j}^{U}\cdot \mathrm{(1}-{\alpha }_{i})$, which is *j*’s reputation discounted by *i*’s prejudice.

Upon comparison, ${r}_{i}^{G}$ is either the smaller reputation (upward self-comparison from *i*), the greater reputation (downward self-comparison from *i*) or the comparison is equal (similarity). The binary variables from *i*'s social comparison heuristic govern whether or not *i* donates in the presence of similarity (*s*_*i*_), upward self-comparison (*u*_*i*_) or downward self-comparison (*d*_*i*_). When $i\in {G}_{{t}_{i},{\alpha }_{i}}$, $|{G}_{{t}_{i},{\alpha }_{i}}|=1$, and *i* is selected to play in-group, no cost is incurred and no donation is made.

The act of donation from agent *i* to *j* results in an economic transaction, with cost *c* to *i* and benefit *b* to *j*. From previous experimentation^25^ we assume a *c*/*b* ratio of 0.7. Within a generation, each agent *i* tracks its accumulation of benefits received less costs from donations (*tp*_*i*_).

### Assessment Rules

Assessment rules are implemented immediately after action rules (i,e., after a donation game between agents *i* and *j*). Both the universal and in-group reputations are updated, based on generalised standing^25^. Standing follows the principle that donations increment reputation, and defection reduces reputation, unless there is a legitimate reason. In this case we adopt the reason to be that an agent determines the recipient’s reputation as less than their own.

Both ${r}_{i}^{U}$ and ${r}_{i}^{G}$ are incremented when the donor *i* cooperates. To update ${r}_{i}^{U}$, if agent *i* defects on *j* then ${r}_{i}^{U}$ is decremented unless ${r}_{j}^{U} < {r}_{i}^{U}$ (i.e., *j* is perceived to be less cooperative and of lower standing), in which case the universal reputation ${r}_{i}^{U}$ remains unchanged. An exactly analogous process is applied to update ${r}_{i}^{G}$ when *i* and *j* are in-group. When *j* is out-group to *i* and agent *i* defects on *j*, then ${r}_{i}^{G}$ is decremented unless ${r}_{j}^{U}\cdot \mathrm{(1}-{\alpha }_{i}) < {r}_{i}^{G}$ (i.e., *j* is perceived to be less cooperative and of lower standing than *i*, while also taking into account *i*’s out-group prejudice). At the beginning of a new generation, the in-group and universal reputations of all agents are reset to zero and reputations are assumed to be public, visible to all agents.

### Selection and Reproduction

Each reproductive step occurs after 5000 randomly selected agent pairs have played the donation game (one generation). To counter potential genetic drift in small sub-populations, an agent’s chance of reproduction, at the reproductive step, is limited to 10%, which is similar to approaches applied in a spatial context (e.g.^15,65^). The reproductive step is repeated after each of 50,000 generations, unless otherwise stated.

At a reproductive step, all agents are considered for reproduction. If agent *i* is successful (10% chance), it copies the social comparison heuristic of another agent; otherwise agent *i* carries forward its current social comparison heuristic to the next generation.

To perform reproduction, the total payoff for an agent during a generation, denoted *tp*_*i*_, is defined as: the total benefit received from donations to *i*, less the total cost *i* has incurred in making donations. We let tp^*^ denote the lowest negative value of total payoff across all agents *i*, in the current generation, with tp^*^ = 0 if such a negative value of tp^*^ doesn’t exist. Then the fitness *f*_*i*_ for an agent *i* during a generation is defined as

1 $${f}_{i}=t{p}^{\ast }+t{p}_{i}+\delta $$

where *δ* is a small constant. Throughout we apply *δ* = 1. Note that tp^*^ and *δ* ensure that fitness is non-zero, while *tp*_*i*_ provides a relative weighting based on payoff.

If agent *i* goes forward to update its social comparison heuristic, then agent *i* selects an agent *j* from which to copy, using relative fitness. Copying takes place from either the in-group (i.e., locally) with probability *P*_*i*_, or from the whole population with probability 1 − *P*_*i*_. This follows the Island model principle^49,66^, with Islands corresponding to prejudicial in-groups. If an agent $i\in {G}_{{t}_{i},{\alpha }_{i}}$ copies a social comparison heuristic from the in-group, then the probability of copying the social comparison heuristic of agent $j\in {G}_{{t}_{i},{\alpha }_{i}}$ is:

2 $$\frac{{f}_{j}}{\sum _{k\in {G}_{{t}_{i},{\alpha }_{i}}}\,{f}_{k}}$$

Alternatively if an agent $i\in {G}_{{t}_{i},{\alpha }_{i}}$ copies a social comparison heuristic from the whole population of agents, denoted *N*, then the probability of copying the social comparison heuristic of agent *j* ∈ *N* is:

3 $$\frac{{f}_{j}}{\sum _{k\in N}\,{f}_{k}}$$

A random mutation to each element of the agent’s new social comparison heuristic (unless, in the case of *S*_*i*_ and *P*_*i*_, they are fixed by the experiment), at the rate of 1% across each variable^25^. This general clonal approach to reproduction is dependent on a single parent and is commonly used in previous studies on indirect reciprocity based on evolutionary simulation^22,24,25,49^.
